# Epigenetic age acceleration in peripheral blood correlates with brain-MRI age acceleration

**DOI:** 10.1093/brain/awaf069

**Published:** 2025-02-24

**Authors:** Pedro Sant'Anna Barbosa Ferreira, Jenny van Dongen, Anouk den Braber, Dorret I Boomsma, Eco J C de Geus, Dennis van ’t Ent

**Affiliations:** Department of Biological Psychology, Vrije Universiteit Amsterdam, 1081 BT Amsterdam, The Netherlands; Department of Biological Psychology, Vrije Universiteit Amsterdam, 1081 BT Amsterdam, The Netherlands; Amsterdam Public Health Research Institute, 1081 HV Amsterdam, The Netherlands; Department of Biological Psychology, Vrije Universiteit Amsterdam, 1081 BT Amsterdam, The Netherlands; Alzheimer Center Amsterdam, Neurology, Vrije Universiteit Amsterdam, Amsterdam UMC, 1081 HZ Amsterdam, The Netherlands; Amsterdam Neuroscience, Neurodegeneration, 1081 HV Amsterdam, The Netherlands; Amsterdam Public Health Research Institute, 1081 HV Amsterdam, The Netherlands; Department of Complex Trait Genetics, Center for Neurogenomics and Cognitive Research, Vrije Universiteit Amsterdam, 1081 HZ Amsterdam, The Netherlands; Amsterdam Reproduction and Development (AR&D) Research Institute, 1081 HV Amsterdam, The Netherlands; Department of Biological Psychology, Vrije Universiteit Amsterdam, 1081 BT Amsterdam, The Netherlands; Amsterdam Public Health Research Institute, 1081 HV Amsterdam, The Netherlands; Department of Biological Psychology, Vrije Universiteit Amsterdam, 1081 BT Amsterdam, The Netherlands; Amsterdam Public Health Research Institute, 1081 HV Amsterdam, The Netherlands

**Keywords:** DNA methylation, brain structure, ageing, association, twin models

## Abstract

As the world’s population ages, more and more people are expected to suffer from age-related diseases. Biological ageing markers derived from DNA methylation and brain structure show promise in predicting health outcomes. Understanding the relationship between these biomarkers can promote the development of effective health interventions.

In a sample of 254 participants from the Netherlands Twin Register (20–84 years), we investigated associations between DNA methylation age acceleration based on five epigenetic biomarkers (Hannum, Horvath, PhenoAge, GrimAge and DunedinPACE) and brain age acceleration based on neuroimaging (brainageR). Furthermore, we applied bivariate twin models to examine the contribution of genetic and environmental factors to the associations (cross-twin cross-trait correlations and within monozygotic-twin pair differences).

We observed relationships with brain age acceleration for DNA methylation age acceleration based on the Hannum and GrimAge clocks that were supported by within monozygotic-twin pair difference modelling. Cross-twin cross-trait modelling confirmed a non-shared environmental aetiology.

Twin analyses highlight the importance of the environment in accelerated ageing, raising the possibility for interventions such as lifestyle modification.

## Teaser

In a cohort of twins, Sant’Anna Barbosa Ferreira *et al.* reveal associations between age acceleration estimates from brain structural MRI and DNA methylation in blood. Their findings emphasize the role of non-shared environmental factors in these associations, underscoring the potential utility of lifestyle interventions.

## Introduction

As the world’s population ages,^1^ more people will experience age-related health complications.^2,3^ However, the rate of biological ageing and associated health outcomes vary from person to person.^4^ While some reach old age in good health with an active lifestyle, others experience health problems and need support already at earlier or similar ages.^4,5^ Over the past decades, several biomarkers have been evaluated for their ability to capture an individual’s age based on biological measurements,^6^ including age-related epigenetic changes due to variation in DNA methylation and age-related changes in brain structure.^7-10^

The first generation of epigenetic age estimators (‘epigenetic clocks’) was developed to predict chronological age.^11,12^ The Hannum clock^13^ and the Horvath clock^14^ are first-generation clocks that are widely used.^11^ The Hannum clock is based on methylation at 71 CpG sites in white blood cells (WBC)^13^ and the Horvath variant is based on methylation at 353 CpG sites in 51 different tissues and cell types.^14^ Recognizing that chronological age is an imperfect proxy for measuring biological age,^11^ a second generation of clocks was developed with physiological phenotypes as targets for predictions.^15^ A clock called PhenoAge^12^ was designed to predict an age measure based on various blood-based biomarkers and chronological age. This clock is based on methylation at 513 relevant CpG sites in WBC. Another second-generation clock, called GrimAge,^16^ was developed by regressing time to death (from any cause) on DNA methylation in seven blood plasma proteins and smoke pack-year. This clock identified 1030 relevant CpG locations. From all these clocks, a measure of age acceleration can be obtained by computing the difference between the clock’s age prediction and actual chronological age. A ‘third-generation’ clock name DunedinPACE was developed to directly predict the pace of ageing, i.e. how quickly a person is ageing at a given moment in time.^17^ Here, the pace of ageing was first determined in every included subject from longitudinal changes in 19 biomarkers, assessing cardiovascular, metabolic, renal, hepatic, immune, dental and pulmonary systems. The clock was then built by regressing the computed pace of ageing on DNA methylation data from blood. This procedure identified 173 relevant CpG sites.

Alternatively, biological age can be derived from brain structure measured with MRI.^7,10^ An algorithm that has been widely used in recent years and shows high prediction accuracy for chronological age is brainageR.^18^ As with the epigenetic clocks, age acceleration can be derived by computing the difference between brain age and chronological age. Accelerated brain ageing estimates by brainageR have shown significant associations with other classical phenotypic indicators of ageing (e.g. weaker grip strength, poorer lung function, lower fluid intelligence), neurological diseases (e.g. dementia) and mortality.^18,19^

To date, only a handful of studies have been able to directly compare estimates of age acceleration based on DNA methylation and brain structure. These studies have focused on different sets of epigenetic clocks (see Supplementary Table 1 for an overview). No associations were observed when DNA methylation age was inferred from the Hannum,^20^ Horvath^18,20-22^ and PhenoAge^20,21^ epigenetic clocks. However, three studies have reported an association when DNA methylation age was inferred from GrimAge.^20,23,24^ This is in accordance with a recent study showing that correlations between different epigenetic clocks do not exceed 0.5^17^ and indicates the relevance of epigenetic clock selection for comparison with brain age.

In the current study, we provide a comprehensive evaluation of how age estimates based on brain structure from MRI compare with the, at present, most studied first-, second- and third-generation epigenetic clocks (Hannum, Horvath, PhenoAge, GrimAge and DunedinPACE) using DNA methylation in blood. As a major innovation, our research in a genetically informative sample consisting primarily of twins, also allowed us to examine the contribution of genetic and environmental factors to the relationships. Variation in DNA methylation is typically depicted as caused by environmental factors but there is evidence supporting a heritable component as well (for an overview see Villicaña and Bell^25^). Likewise, environmental and genetic factors contribute to variation in brain structure.^26^ Thus, the associations between age acceleration at the epigenome level and age acceleration at the brain level may be explained by genetic and environmental factors that influence both ageing-related processes.

## Materials and methods

### Participants

The subjects in this study took part in MRI studies of the Netherlands Twin Register (NTR) and the Netherlands Twin Register biobank project.^27^ MRI data were available for 671 monozygotic (MZ), dizygotic (DZ) twins, and additional siblings of twins registered with the NTR^27,28^ who had participated in one of five different MRI twin studies (see Table 1). Good quality whole-blood DNA methylation data were available for 3089 samples from 3057 individuals from twin families,^34^ of which 3087 were analysed in the current study (two individuals who retracted permission for data use were removed); 276 individuals had both MRI data and DNA methylation data.

**Table 1:** Participant’s biological measures per MRI study

	MRI original study				
Measures	S1	S2	S3	S4	S5
**Complete sample**					
Males/females, *n*	28/46	93/150	26/39	83/114	0/92
Age at MRI, years	14.76 ± 1.41	33.62 ± 9.84	29.78 ± 7.79	70.60 ± 7.54	47.16 ± 8.15
Age range (MRI), years	11–18	19–57	20–42	60–94	30–71
**MRI with blood subsample**					
Males/females	0/4	46/84	15/18	31/39	0/43
Age at MRI, years	14.50 ± 0.58	37.95 ± 8.56	30.22 ± 5.76	68.08 ± 6.91	47.58 ± 9.57
Age range (MRI), years	14–15	23–57	20–40	60–84	30–71
Age at blood collection, years	20.15 ± 2.14	35.03 ± 8.41	33.55 ± 6.27	59.21 ± 6.44	39.60 ± 9.83
Age range (blood), years	18–22	21–53	25–45	50–75	21–64
Δ Age (MRI-blood), years	−5.65	2.92	−3.33	8.87	7.98
Neutrophils, %	56.13	53.07	50.67	50.97	54.78
Monocytes, %	8.48	8.48	8.50	9.04	8.30
Eosinophils, %	1.18	3.07	3.15	3.48	2.53
Lymphocytes, %	34.10	34.92	37.10	36.01	33.94
Basophils, %	0.13	0.45	0.58	0.51	0.46
BMI, kg/m^2^	20.36	23.56	24.09	25.63	24.39
Smoking status (N/F/C), *n*	4/0/0	84/22/24	28/03/02	30/27/13	22/15/6

S1 = MRI study 1 by van ’t Ent *et al*.^29^; S2 = MRI study 2 by den Braber *et al*.^30^; S3 = MRI study 3 by de Geus *et al*.^31^; S4 = MRI study 4 by Konijnenberg *et al*.^32^; S5 = MRI study 5 by Doornweerd *et al*.^33^; smoking status (N/F/C) = non-smoker, former smoker and current smoker.

All participants (or parents in the case of children) provided written informed consent. The studies were approved by the Central Ethics Committee on Research Involving Human Subjects of the VU University Medical Centre, Amsterdam, an Institutional Review Board certified by the U.S. Office of Human Research Protections (IRB number IRB00002991 under Federal-wide Assurance- FWA00017598; IRB/institute codes, NTR 2003-61, 2005-246, Po3.18ooC, 2013-263; IRB MRI study 4: 2014.210).

### Brain MRI

MRI study 1 by van ’t Ent *et al*.^29^ provided data from 68 MZ twins (34 complete twin pairs), and six DZ twins (three complete pairs). The adolescent twins were selected to be concordant or discordant for ratings on the Child Behaviour Checklist Attention Problem scale (CBCL-AP). MRI was obtained using a Siemens Sonata 1.5 T scanner (Siemens) with a standard circularly polarized head coil. A 3D magnetization prepared rapid gradient echo (MPRAGE) T1-weighted sequence was run for all participants. Each volume consisted of 160 sagittal slices (1.00 × 1.00 × 1.00 mm), with an in-plane voxel size of 1 mm^2^ [repetition time (TR) = 1900 ms; inversion time (TI) = 1100 ms; echo time (TE) = 3.93 ms; flip angle (FA) = 15° and 256 × 224 matrix].

MRI study 2 by den Braber *et al*.^30^ contributed data from 140 MZ twins (68 complete pairs), 97 DZ twins (47 complete pairs) and six siblings. Twin pairs were selected based on an age range between 18 and 60 years and discordant, concordant-high or concordant-low scores for obsessive–compulsive symptoms. MRI was collected using a Philips Intera 3.0 Tesla scanner (Philips, Medical Systems) with a standard sensitivity encoding (SENSE) receiver head coil. A 3D gradient-echo T1-weighted sequence (technique: T1TFE) was run for all participants. Each volume consisted of 182 coronal slices (1.00 × 1.00 × 1.20 mm), with an in-plane voxel size of 1 mm^2^ (TR = 9.64 ms, TE = 4.60 ms, FA = 8° and 256 × 256 matrix).

MRI study 3 by de Geus *et al*.^31^ contributed data from 59 MZ twins (29 complete pairs) and six DZ twins (three complete pairs). Selection criteria included age ranging between 18 and 50 years and discordant or concordant anxious depression scores. MRI acquisition was performed using a Siemens Sonata 1.5 T scanner (Siemens) with a standard circularly polarized head coil. A 3D MP-RAGE T1-weighted sequence was run for all participants. Each volume consisted of 160 coronal slices (1.00 × 1.00 × 1.50 mm), with an in-plane voxel size of 1 mm^2^ (TR = 15 ms, TI = 300 ms, TE = 7.00 ms, FA = 8° and 256 × 176 matrix).

MRI study 4 by Konijnenberg *et al*.^32^ provided data from 195 MZ twins (95 complete twin pairs), two DZ twins (one complete pair). Participants were only included if they were 60 years or older and cognitively normal (Clinical Dementia Rating Score of zero). MRI acquisition was performed using a 3.0 T Philips Achieva scanner using an 8-channel head coil. The MRI protocol included 3D-T1 with sagittal turbo field echo sequence (1.00 mm × 1.00 mm × 1.00 mm voxels, TR = 7.9 ms, TE = 4.5 ms, FA = 8°).

MRI study 5 by Doornweerd *et al*.^33^ provided data from 33 MZ twins (16 complete twin pairs), 34 individual DZ twins and 25 twins without zygosity information. MRI acquisition was performed on a 3.0 T GE Signa Hdxt scanner (General Electric). T1 weighted scans were acquired using a 3D fast spoiled gradient-echo sequence (TR = 2160 ms, TE = 30 ms, FA = 80°, slice thickness 3 mm, matrix size 64 × 64, 211 × 211 mm^2^ field of view, voxel size 3 × 3 × 3 mm, 40 slices).

Blood DNA methylation data (Illumina 450k arrays) were available for a subset of the participants: 262 MZ twins (120 complete pairs) and 85 DZ twins (32 complete pairs). Age (range) and male/female ratio for the MRI dataset and the MRI with blood dataset is depicted in Table 1, as well as the time between MRI and blood sampling [Δ Age (MRI-blood)].

### Biological age from brain structure

In the total MRI datasets combined (*n* = 617), brain age was calculated from the structural MRI scans using the brainageR method.^18^ At this step, we decided to exclude 73 participants because their ages were outside the 18–92 year age range for which brainageR was trained. In the final sample (*n* = 598), age estimates based on brain structure were significantly correlated with chronological age (see Supplementary Fig. 1: Pearson *r* = 0.93, *P* < 0.001). The root mean square error (RMSE) and mean absolute error (MAE) for the predicted brain age versus chronological age were 7.10 years and 5.49 years, respectively. Brain age acceleration was obtained by subtracting chronological age from estimated brain age. Given age-related bias in brain age estimation models,^35^  ^,36^ a sample-level correction was performed on the age acceleration estimates using Beheshti’s method.^37^

### Biological age from DNA methylation

Blood collection procedures are described in Willemsen *et al*.^27^ The Infinium Human Methylation 450 Bead Chip Kit (Illumina) was used to measure DNA methylation at the Human Genotyping Facility of the Erasmus MC, The Netherlands.^38^ Procedures for DNA methylation profiling, quality control and normalization are described by van Dongen *et al*.^34^ In the total DNA methylation dataset (*N* = 3087), epigenetic age estimates were computed from DNA methylation using Horvath epigenetic age calculator software^14^ and the R package DunedinPACE.^17^ Estimates for age acceleration indexed by Hannum, Horvath, PhenoAge and GrimAge were obtained by taking the residuals of the regression of estimated biological age from DNA methylation on chronological age. The corresponding output variables of Horvath’s epigenetic age calculator are: AgeAccelerationResidualHannum (AARH), Intrinsic Epigenetic Age Acceleration (IEAA), AgeAccelPheno (AAPheno) and AgeAccelGrim (AAGrim).

The performance of DNA methylation age estimates (see Supplementary Figs 2–5) was evaluated in our total sample of participants with Illumina 450k array DNA methylation data from blood samples (*n* = 3087) with Pearson’s *r*, the RMSE and MAE. Correlations ranged from 0.92 (PhenoAge) to 0.94 (Horvath). MAE ranged from 3.2 years (Horvath) to 9.3 years (Hannum) and RMSE ranged from 4.2 years (Horvath) to 10.1 years (Hannum).

### Statistical analyses

All analyses were conducted in R.^39^ Associations between standardized brain and DNA methylation age acceleration were estimated with linear mixed effects models to account for family relatedness in our sample. First, we applied a base model, in which brain age acceleration was regressed once on each measure of DNA methylation age acceleration inferred by each epigenetic clock (Hannum, Horvath, PhenoAge, GrimAge, DunedinPACE) controlled for bisulphite sample plates (dummy-coding) and methylation array row number. To account for the small number of observations within (some of) the original sample plates, bisulphite sample plates were recoded such that the 34 original plates were reduced to six variables by collapsing consecutive plates. Next, we recomputed a full model with additional covariates for sex, BMI and smoking status (former smoker versus non-smoker and current smoker versus non-smoker), along with measured percentage counts of WBC to account for variations in blood cell composition.^40^ For WBC, only the percentage counts of monocytes, eosinophils and neutrophils were considered. Lymphocyte counts were not included due to a high correlation with neutrophils (r = −0.95, *P* < 0.001), and basophil counts were not included because they showed little variation between individuals (*M* = 0.46, SD = 0.65, median = 0.30).

From our initial sample, we removed eight individuals with missing values for covariates and another 14 based on Rosner’s test^41^ for outliers (see Supplementary Table 2). This left a final sample of 254 individuals with brain and DNA methylation age acceleration data (see Supplementary Fig. 6). We transformed all continuous variables to *Z*-scores, thus, all estimates for association (betas) can be interpreted as correlations.

As an additional analysis step, to examine the nature of the association between brain and DNA methylation age acceleration we computed cross-twin cross-trait (CTCT) correlations.^42^ This was necessarily limited to the MZ twin pairs, as the number of complete DZ pairs was very small (*n* = 9). If partly the same genetic factors, and/or shared environmental factors, influence DNA methylation and brain age acceleration, we expect the CTCT correlation in MZ twins to be significantly larger than zero, meaning that the brain age acceleration in a twin can predict the DNA methylation age acceleration in the co-twin.

Finally, to test if the association between brain and DNA methylation age acceleration is caused by unique person-specific environmental factors influencing these two traits, we regressed the within-MZ pair differences in brain age acceleration on the within-MZ pair differences in DNA methylation age acceleration (ΔMZTwin).^43^ Unique environmental effects would give rise to a correlation of the MZ intra-pair differences in brain and DNA methylation age acceleration, which could not be caused by genetic or shared environmental differences in MZ pairs as these are absent.

To account for multiple testing, we applied a Bonferroni correction based on the number of independent tests. A prior study by our group^44^ identified three independent dimensions among the five epigenetic biomarkers used in the current analysis (Hannum, Horvath, PhenoAge, GrimAge and DunedinPACE). Therefore, we set the significance threshold at 0.0167 (α = 0.05/3) for this study.

### Sensitivity analyses

In the context of sensitivity analyses, we recomputed the full model including MRI scanner field strength as an additional covariate to control for potential differences in brain age estimates due to scanner strength.^45^ To test for a possible influence of the time between MRI and blood collection, we also applied our full model (all covariates) to two subgroups. The first subgroup was limited to all individuals who had blood drawn more than 2 years before the time of the MRI scan while the second was limited to those who had blood drawn more than 4 years before the time of the MRI scan.

## Results

### Association between brain and DNA methylation age acceleration

Results of the regressions of brain age acceleration on DNA methylation age acceleration summarized in Table 2 indicated positive correlations between brain age acceleration and DNA methylation age acceleration based on the Hannum and GrimAge epigenetic clocks in both the base model and the full model with added covariates. Only Hannum was significant in both models after correction for multiple testing. The full model regressions indicate an effect of sex with higher brain age acceleration observed in males (Hannum: b = 0.38, *P* = 0.009; GrimAge: b = 0.38, *P* = 0.011).

**Table 2:** Associations of DNA methylation age acceleration with brain age acceleration

			Linear mixed models (*n* = 254)															
	Base model		Full model															
	DNAmAA		DNAmAA		Sex		BMI		Former smoker		Current smoker		Eosinophils		Monocytes		Neutrophils	
Epiclock	b	*P*	b	*P*	b	*P*	b	*P*	b	*P*	b	*P*	b	*P*	b	*P*	b	*P*
Hannum	0.14	0.011^b^	0.15	0.011^b^	0.38	0.009^b^	−0.09	0.137	0.07	0.600	0.15	0.335	0.13	0.024^a^	−0.06	0.341	−0.07	0.219
Horvath	−0.01	0.845	−0.02	0.780	0.44	0.003^b^	−0.08	0.194	0.06	0.646	0.20	0.228	0.13	0.030^a^	−0.05	0.463	−0.04	0.554
PhenoAge	−0.03	0.572	−0.02	0.735	0.43	0.004^b^	−0.08	0.197	0.06	0.643	0.20	0.226	0.13	0.028^a^	−0.05	0.482	−0.03	0.649
GrimAge	0.17	0.005^b^	0.16	0.035^a^	0.38	0.011^b^	−0.07	0.243	−0.03	0.838	−0.05	0.801	0.13	0.033^a^	−0.03	0.682	−0.06	0.318
DunedinPACE	−0.01	0.925	0.03	0.698	0.45	0.003^b^	−0.08	0.168	0.05	0.718	0.17	0.327	0.13	0.030^a^	−0.04	0.486	−0.04	0.497

Base model, brain age acceleration regressed on DNA methylation age acceleration (DNAmAA) and technical covariates (bisulphate sample plates and array methylation row number); full model, brain age acceleration regressed on DNA methylation age acceleration (DNAmAA), technical covariates, body mass index (BMI), smoking status and white blood cell percentage counts. epiclock = epigenetic clock.

^a^ *P* < 0.05 (Nominal).

^b^ *P* < 0.0167 (Bonferroni).

### Cross-twin cross-trait analyses

CTCT analysis (see Table 3, column ‘CTCT’) indicated no significant associations between DNA methylation age acceleration and brain age acceleration.

**Table 3:** Genetic and environmental models performed in the full sample

	CTCT			ΔMZTwin		
Epiclock	*n*	r	*P*	*n*	b	*P*
Hannum	178	−0.05	0.593	89	0.17	0.030^a^
Horvath	178	−0.02	0.826	89	−0.05	0.533
PhenoAge	178	−0.11	0.183	89	−0.08	0.287
GrimAge	178	0.07	0.407	89	0.25	0.006^b^
DunedinPACE	178	−0.08	0.373	89	0.09	0.384

CTCT = cross-twin cross-trait model; ΔMZTwin = MZ twin difference model.

^a^ *P* < 0.05 (Nominal).

^b^ *P* < 0.0167 (Bonferroni).

### MZ twin difference modelling

**Figure 1:**
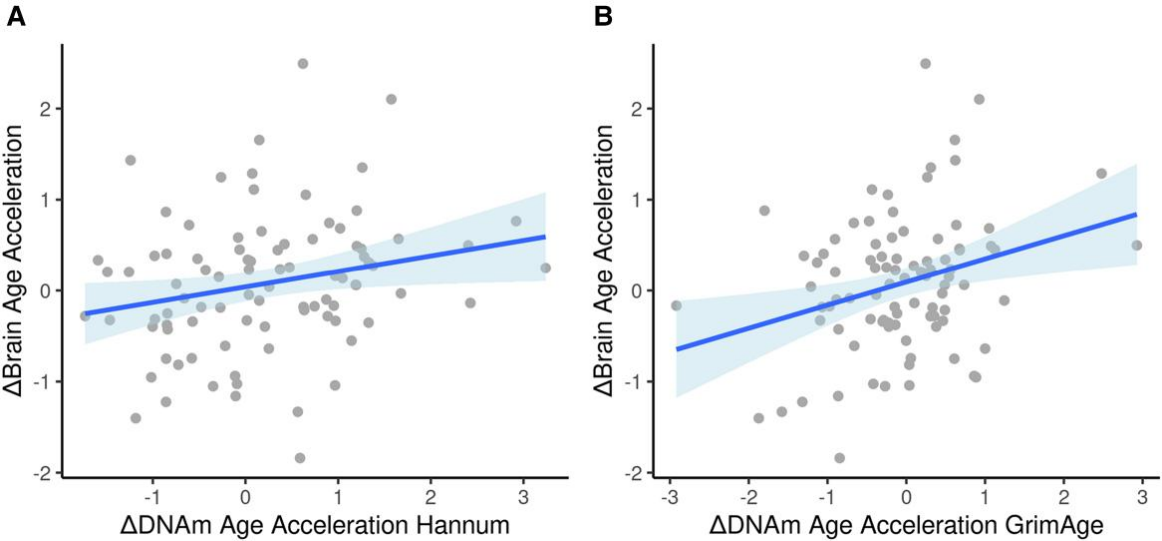
**Associations between within MZ twin pair differences for brain age acceleration (ΔBrain age acceleration) and DNA methylation age acceleration (ΔDNA methylation age acceleration) measures (A) based on Hannum and (B) based on GrimAge**. Blue line indicates model fit. Blue shadow indicates confidence interval. Units are in standardized years.

Modelling MZ twin differences in the full sample (see Table 3, column ‘ΔMZ Twin’) indicated positive associations for differences in brain age acceleration with differences in DNA methylation age acceleration for both the Hannum and the GrimAge clocks, which was statistically significant for GrimAge (see Fig. 1).

### Sensitivity analyses

Our sensitivity analysis revealed no significant effects related to differences in MRI scan field strength (Hannum: b = 0.31, *P* = 0.195; GrimAge: b = 0.33, *P* = 0.164).

In addition, we repeated our full regression model in two subsets of our sample considering the difference between the date of blood draw and the date of the MRI scan. The results indicated trends in line with our main analyses (see Table 4), albeit no longer statistically significant as expected given the reduction in sample size. Point estimates of similar magnitude were observed for both Hannum and GrimAge when the time of blood collection was more than 2 years or more than 4 years prior to the MRI scan.

**Table 4:** Associations of brain age acceleration with DNA methylation age acceleration in subsets where blood collection was prior to the MRI scan

	Linear mixed model					
	Regression model: 2 years prior			Regression model: 4 years prior		
Epiclock	*n*	b	*P*	*n*	b	*P*
Hannum	185	0.13	0.047^a^	133	0.13	0.120
Horvath	185	−0.03	0.712	133	0.02	0.841
PhenoAge	185	−0.08	0.224	133	−0.09	0.311
GrimAge	185	0.14	0.110	133	0.17	0.139
DunedinPACE	185	0.07	0.432	133	0.08	0.446

^a^ *P* < 0.05 (Nominal).

## Discussion

We examined associations between biological age acceleration from MRI-derived brain structure and from DNA methylation (DNA methylation) in blood cells using five different epigenetic clocks (Hannum, Horvath, PhenoAge, GrimAge and DunedinPACE). The results, across a broad age range (20–84 years), indicated weak but significant positive relationships for brain age acceleration with DNA methylation age acceleration from Hannum and GrimAge clocks, with effect sizes equivalent to correlations of around 0.15.

Associations between brain age acceleration and DNA methylation age acceleration indexed by GrimAge have been noted in two previous studies (see Supplementary Table 1). McLachlan *et al*.^23^ observed an association between these two measures in a study investigating a potential relation between attitudes to ageing and age acceleration biomarkers. The sample consisted of 758 adults, with a mean age of 72.5 years, from the Lothian Birth Cohort 1936. Peterson *et al*.^24^ reported comparable findings when exploring a potential correlation between the two metrics and chronic pain. Their study involved 174 individuals with a mean age of 57.9 years. In further support, a recent study reported an association between hippocampal volume reduction and DNA methylation age acceleration indexed by Hannum^46^ in individuals with Alzheimer’s disease-related neuroimaging phenotypes (volumetric MRI and amyloid-β PET) of two Australian cohort studies [the Australian Imaging Biomarkers and Lifestyle (AIBL) study^47^ and Alzheimer's Disease Neuroimaging Initiative (ADNI)^48^]. A significant association was observed in the AIBL cohort, a sample of 329 individuals with a mean age of 73.4 years. The study also examined other epigenetic clocks, namely Horvath, Zhang and PhenoAge, and found significant results when using PhenoAge.

The mechanisms linking blood-based epigenetic age acceleration to brain age acceleration remain unclear. The Hannum clock, a first-generation epigenetic clock, was developed to measure immune system ageing and, like brainageR, was trained on chronological age. In contrast, GrimAge was designed as a biomarker to predict time to death and is strongly influenced by health-related factors such as cigarette smoke exposure. Our findings of associations with these two epigenetic biomarkers, but not others, suggest that brain age acceleration may reflect a shared component with age acceleration captured by the Hannum and GrimAge clocks measured in circulating WBC. A potential explanation for the specific correlation between brain age acceleration and Hannum DNA methylation age acceleration could be the shared training on chronological age. However, this does not fully account for the lack of association with the Horvath clock.

One tentative mechanism underlying the associations between brain age and blood-based epigenetic age could be related to inflammatory processes. Blood–brain barrier permeability increases with chronological age,^49,50^ with systemic inflammation over time cited as a major contributing factor.^51^ The damaging effect of inflammation has been associated with the transmigration of leucocytes^52^ and with the overexpression of C-reactive proteins and cytokines.^53^ DNA methylation in leucocytes was used to develop the Hannum clock.^13^ Furthermore, C-reactive proteins and cytokines are regulators of tissue inhibitor metalloproteinases (TIMPs)^50^ and TIMP-1 is one of the DNA methylation-based markers used to engineer GrimAge.^16^ We were unable to test this hypothesis directly, as our analyses did not include inflammation markers. However, previous studies have observed positive associations of C-reactive protein concentrations with brain age acceleration^54^ and with DNA methylation age acceleration, both for the Hannum clock^55^ and the GrimAge clock.^56^

Significant associations between brain age acceleration and age acceleration based on the Horvath, PhenoAge and DunedinPACE clocks were absent. This replicates previous studies (Horvath;^18,20,22^ PhenoAge;^20,21^ DunedinPACE^20,22^—see Supplementary Table 1). As mentioned, like Hannum, the Horvath clock is trained to predict chronological age. However, the Horvath clock is constructed based on methylation in different tissues and is regarded to capture a system-wide component of age acceleration that is detectable in all tissues.^14^ PhenoAge and DunedinPACE models have been trained to predict the functional state of many organ systems and tissues.^12,15,17^

Overall, the modest correlations between estimated brain and epigenetic age acceleration observed in this study suggest that the brain and epigenetic age clocks, based on different methods and derived using different training approaches, may capture partially overlapping but also distinct dimensions of ageing, as well as potential measurement error. Our results highlight the need for continued refinement of these clocks and further investigation into which biomarkers most accurately capture age acceleration.

Our sample of twins provided the unique opportunity to explore the aetiology of the associations. CTCT modelling provided little evidence for genetic and shared environmental contributions. Intra-pair MZ twin difference modelling provided clear evidence for an influence of unique environmental factors on the association of brain- with Hannum-based and of brain- with GrimAge-based age acceleration. Together these twin modelling results point to an aetiology that is explained by unique environment. The associations could reflect independent effects of the exposome on both age acceleration traits, but they could also signal causality, e.g. such that effects of DNA methylation age acceleration mechanistically caused changes in brain age acceleration. Previous studies demonstrated that higher levels of BMI, smoking and alcohol consumption were associated with age acceleration based on either DNA methylation^16,57,58^ or changes in brain structure.^36,59^

A limitation of our study was that the age at the MRI scan differed from the age at DNA collection in many participants. We note, however, that analyses segregating the sample in subsets where the time of blood collection occurred more than 2 or more than 4 years prior to the time of the MRI scan pointed to similar results. In both subsets, effect sizes for all measures were in the same direction and of comparable magnitude to the main analyses. Another limitation of our study is the heterogeneity in MRI field strength across the five studies included in our sample, which could potentially influence brain age estimates. Notably, Franke and Gaser^45^ observed higher standard deviations in brain age estimates derived from 1.5 T scans compared with 3.0 T scans, although the estimates remained highly correlated (*r* = 0.91). In addition, in our sensitivity analyses we included MRI field strength as a covariate and found no significant differences in results, suggesting that this variability did not meaningfully impact our findings. Another consideration is the relatively small sample size of 254 participants, with measures of both brain age acceleration and DNA methylation age acceleration, resulting in restricted statistical power to detect associations. The expected associations from the literature (see Supplementary Table 1) are all relatively small. We further note that our twin analyses were restricted to MZ twins. Accordingly, we could not distinguish between genetic and shared environment influences in the CTCT modelling. Finally, we acknowledge that the age-acceleration measures in our study are cross-sectional and do not represent direct measures of ageing which would require longitudinal measurements within individuals over time.

## Conclusion

In conclusion, our study provides evidence for associations between estimates of age acceleration based on structural changes in the brain and DNA methylation in the peripheral blood. This opens the opportunity to further explore the option of blood-based screening tools to identify adverse neurodegenerative outcomes due to biological age acceleration. The underlying mechanisms of the association require further investigation. Our analyses in monozygotic twins highlight the importance of the exposome in age acceleration, opening the possibility for interventions such as lifestyle modification. Uncovering how and in which conditions lifestyle implicates in the association of these biological age acceleration measures is a potential goal for future research.

## Supplementary Material

awaf069_Supplementary_Data

## Data Availability

Netherlands Twin Register (NTR) data can be requested by bona fida researchers (https://ntr-data-request.psy.vu.nl/).
