# Supplementary material for: Epigenetic age acceleration in peripheral blood correlates with brain-MRI age acceleration
Source: Brain. 2025 Feb 24;148(8):2861–8. doi: 10.1093/brain/awaf069 (PMC12316020; doi:10.1093/brain/awaf069)
Supplement: awaf069_Supplementary_Data [file awaf069_supplementary_data.pdf]

# Supplementary Materials

**Table S1. Previous Studies on the relationship between MRI-derived brain age and blood epigenetic clocks**

| Study | N | Epigenetic clock | Brain Age | Findings |
|---|---|---|---|---|
| Cole et al. (2018)[1] | 620 | Horvath's | brainageR | No association (rho = – .007, p = .85) [b c] |
| McLachlan et al. (2020)[2] | 622 | GrimAge | brainageR | Association (r = .198, p < .001) [b c] |
| Teeuw et al. (2021)3[3] | 172 | Horvath's | Schnack et al. (2016) | No association (r = .02, p = .874) [a b d] |
| | 172 | PhenoAge | Schnack et al. (2016) | No association (r = .03, p = .747) [a b d] |
| Peterson et al. (2022)[4] | 174 | GrimAge | brainageR | Association (r = .198, p = .010) [b d] |
| Zheng et al. (2022)5[5] | 326 | Hannum's | SPARE-BA | No association [a c e] |
| | 326 | Horvath's | SPARE-BA | No association [a c e] |
| | 326 | PhenoAge | SPARE-BA | No association [a c e] |
| | 326 | GrimAge | SPARE-BA | Association (r = .04 to .18) [a c e] |
| Sanders et al. (2022)[6] | 386 | Horvath's | Han et al. (2021) | No association (β = -0.60, p > .050) [a b c d e] |
| | 386 | Zhang et al. (2019) | Han et al. (2021) | No association (β = -0.07, p > .050) [a b c d e] |
| | 386 | Shireby et al. (2019) | Han et al. (2021) | No association (β = -0.00, p > .050) [a b c d e] |
| | 386 | PoAm | Han et al. (2021) | No association (β = 0.17, p > .050) [a b c d e] |
| Phyo et al. (2024)[7] | 560 | Hannum's | brainageR | No association (r = - .06, p > .050) [b c e] |
| | 560 | Horvath's | brainageR | No association (r = - .02, p > .050) [b c e] |
| | 560 | PhenoAge | brainageR | No association (r = - .01, p > .050) [b c e] |
| | 560 | GrimAge | brainageR | No association (r = .01, p > .050) [b c e] |
| | 560 | GrimAge2 | brainageR | No association (r = - .01, p > .050) [b c e] |
| | 560 | DunedinPACE | brainageR | No association (r = - .02, p > .050)[) b c e] |

Note. PoAM = Pace of Ageing Methylation (earlier version of DunedinPACE), SPARE-BA = Spatial Patterns of Abnormality for Recognition[8], GrimAge2 = Grim age version 2[9]

[a] Comparison based on brain age acceleration
[b] Comparison based on brain-PAD
[c] Comparison based on DNAm age acceleration
[d] Comparison based on DNAm age difference (DNAm age – chronological age)
[e] No p-values informed (only significant or not significant at p < .050, p < .010, or p < .001

**Table S2 Outliers detected for Biological Age Acceleration Measures, percentage count of White Blood Cells, and BMI**

| Subject | MAge | BAge | MRI Study | Sex | Zygosity | Plate | Array | SS | Outlier (Value) |
|---|---|---|---|---|---|---|---|---|---|
| 16 | 51.0 | 46.5 | den Braber et al.[10] | 0 | 1 | 8 | 3 | Former | IEAA (-17.4) |
| 29 | 67.3 | 57.8 | Konijnenberg et al.[11] | 0 | 1 | 21 | 4 | Current | Mono (16.2) |
| 257 | 39.0 | 34.3 | den Braber et al.[10] | 1 | 2 | 10 | 6 | Current | Eos (17.4) |
| 281 | 82.8 | 72.0 | Konijnenberg et al.[11] | 0 | 1 | 3 | 3 | Non | IEAA (-16.5) |
| 320 | 41.0 | 37.5 | den Braber et al.[10] | 0 | 1 | 10 | 1 | Non | BMI (29.6) |
| 367 | 41.0 | 38.5 | den Braber et al.[10] | 0 | 1 | 5 | 4 | Non | BMI (26.8) |
| 415 | 62.0 | 53.3 | Konijnenberg et al.[11] | 0 | 1 | 26 | 2 | Former | AAGrim (13.6) |
| 436 | 35.0 | 32.1 | den Braber et al.[10] | 0 | 1 | 20 | 2 | Non | Eos (14.9) / Neut (6.9) |
| 480 | 73.6 | 63.3 | Konijnenberg et al.[11] | 0 | 1 | 13 | 5 | Current | Mono (15.9) |
| 505 | 28.0 | 24.3 | den Braber et al.[10] | 1 | 1 | 9 | 5 | Former | Eos (9.0) |
| 573 | 69.6 | 60.5 | Konijnenberg et al.[11] | 1 | 1 | 25 | 2 | Former | IEAA (-14.9) |
| 642 | 26.0 | 22.2 | den Braber et al.[10] | 1 | 1 | 6 | 5 | Non | AARH (-18.4) |
| 671 | 33.0 | 28.0 | den Braber et al.[10] | 1 | 2 | 13 | 1 | Current | AAGrim (14.4) |
| 677 | 25.0 | 25.3 | den Braber et al.[10] | 1 | 1 | 6 | 5 | Non | brainAAc (27.2) |

Note. Mage = Age at MRI scan, BAge = Age at blood collection, MRI Study = Original MRI study, Sex coded as Females (0) and Males (1), Zygosity coded as Monozygotic twin (1) and Dizygotic twin (2), SS = Smoking Status, Current = Current smoker, Former = Former smoker, Non = Non-smoker, Eos = percentage count of Eosinophils, Neut = percentage count of Neutrophils, AARH = Age Acceleration Residual Hannum, IEAA = Intrinsic Epigenetic Age Acceleration, AAPheno = Age Acceleration PhenoAge, AAGrim = Age Acceleration GrimAge, PACE = Dunedin Pace of Aging, brainAAc = brainAge Acceleration

**Figure S1 Association between chronological age and estimated brain age inferred by brainageR**. Grey dots represent individuals. Blue line indicate model fit. Blue shadow indicates confidence interval. Dashed line represents identity

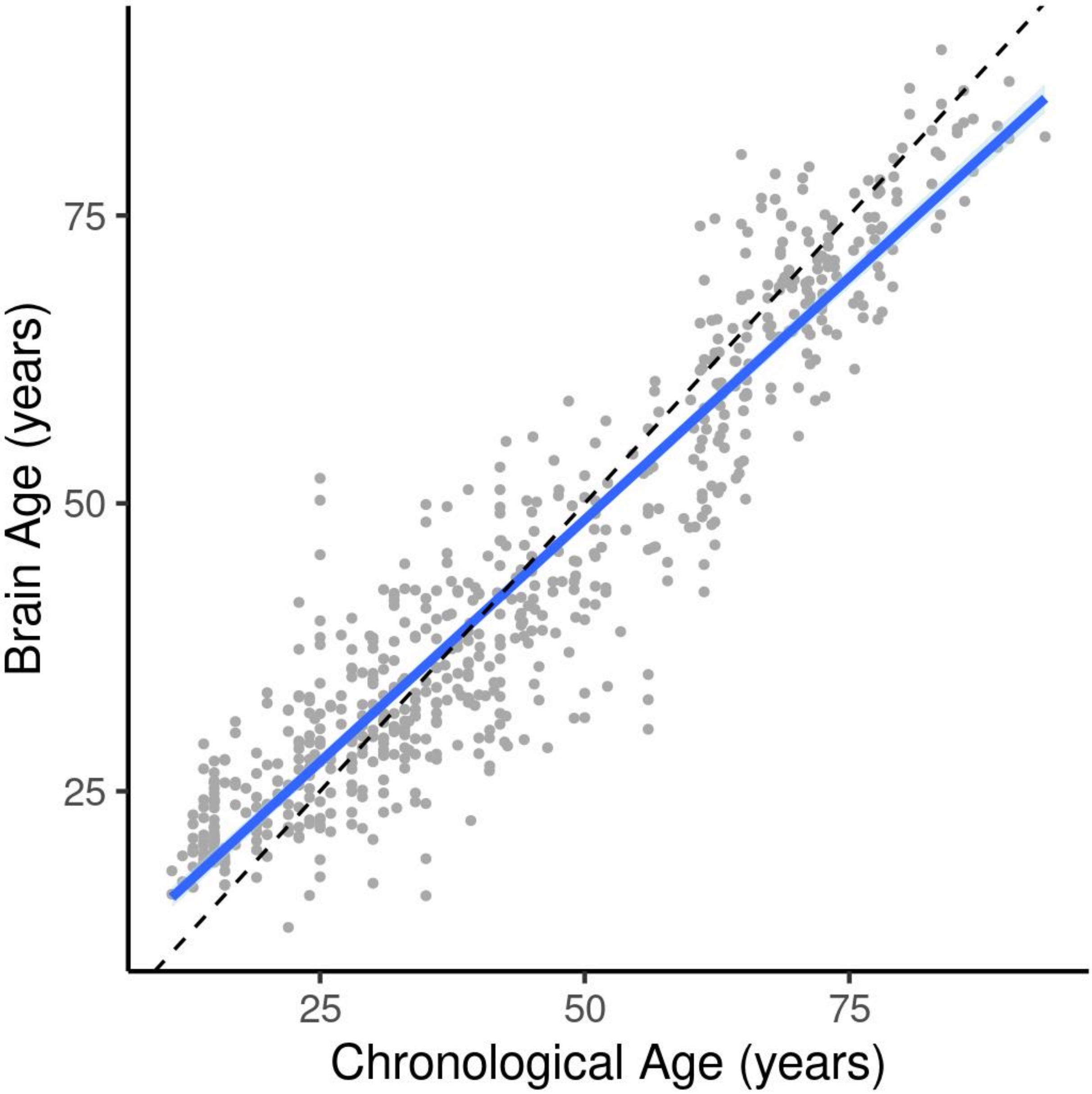

**Figure S2 Association between chronological age and estimated DNA methylation Age inferred by Hannum.** Black dots represent individuals. Red dashed line indicates model fit. Blue dashed line indicates identity line

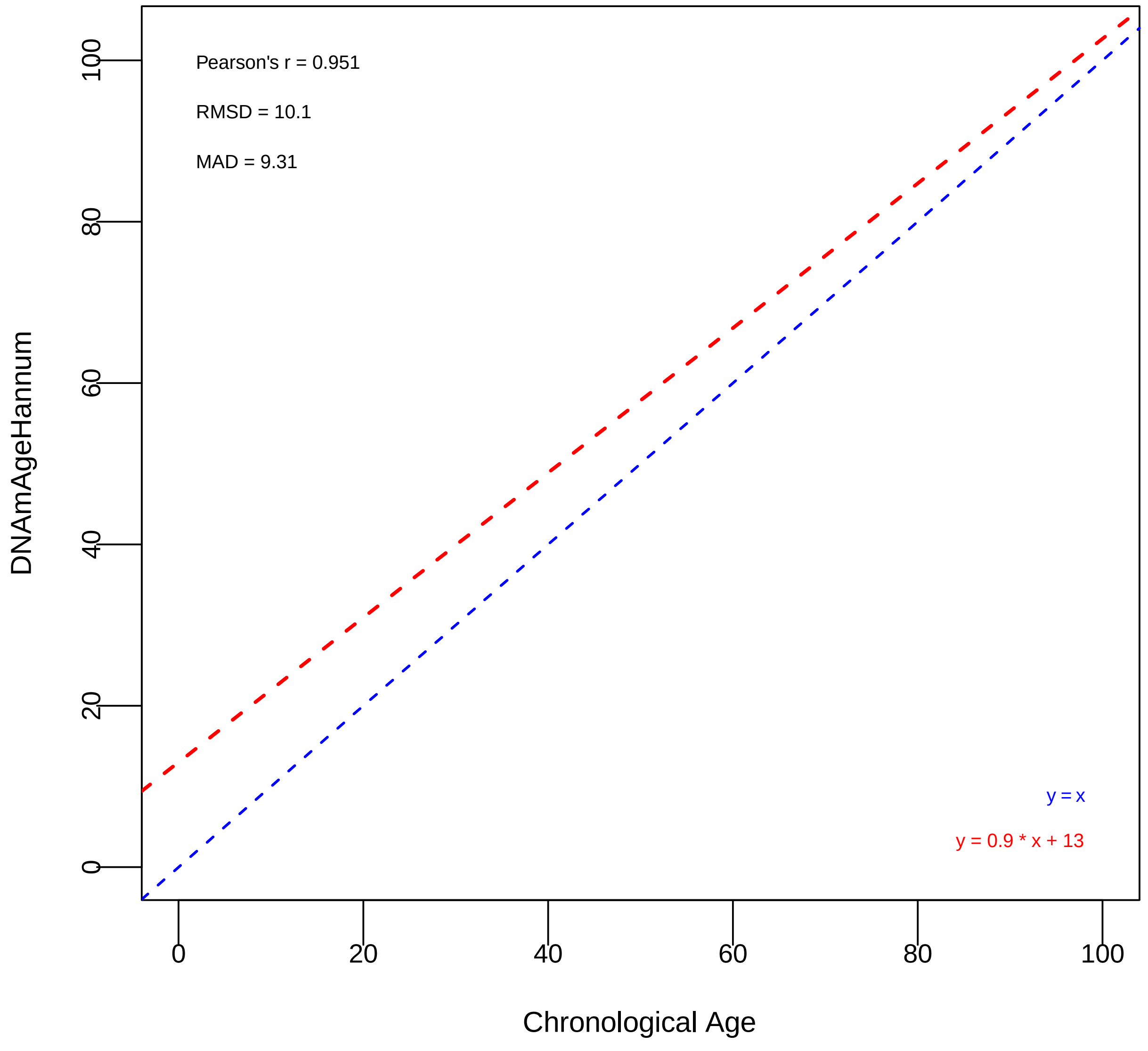

**Figure S3 Association between chronological age and estimated DNA methylation Age inferred by Horvath.** Black dots represent individuals. Red dashed line indicates model fit. Blue dashed line indicates identity line

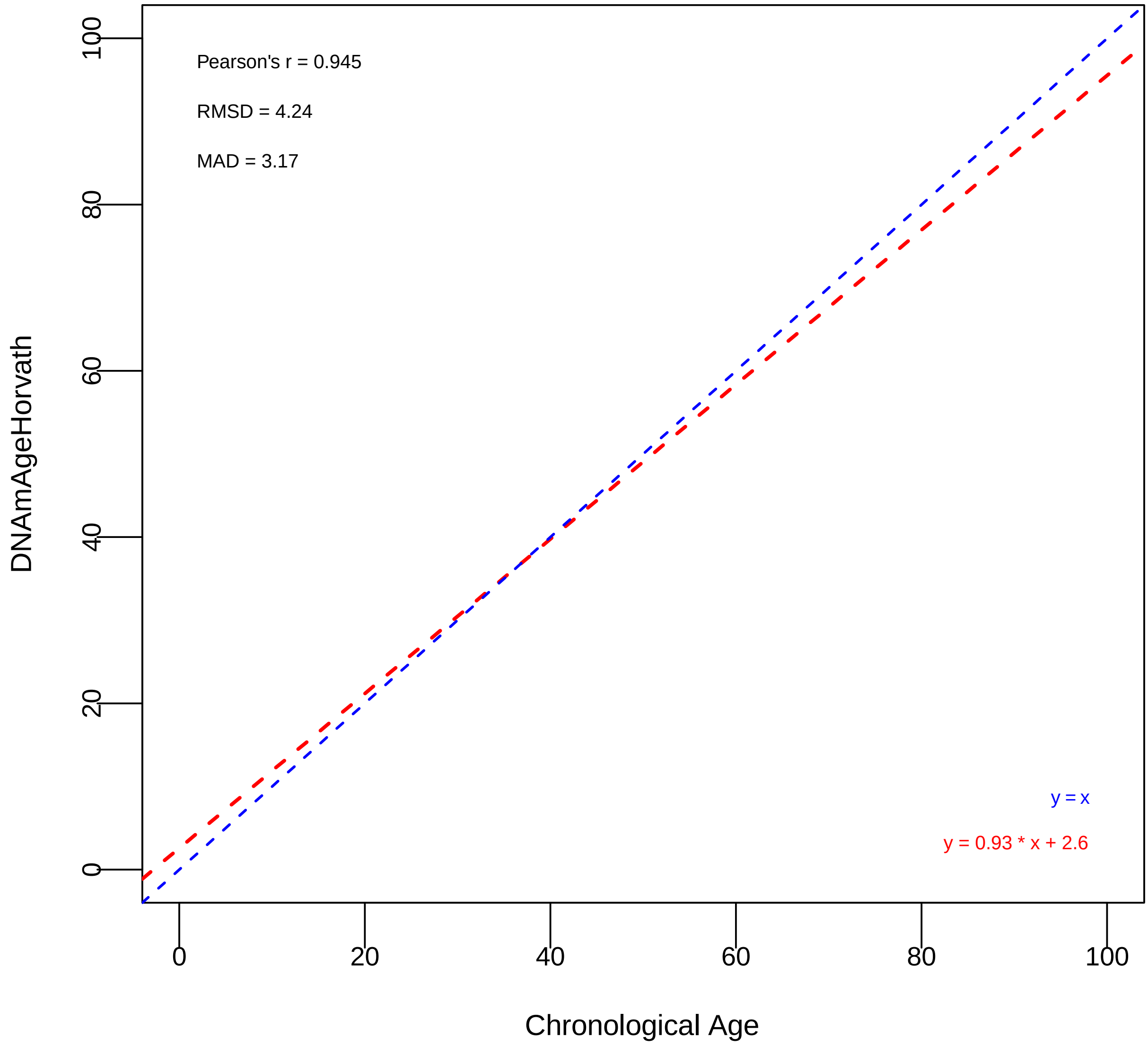

**Figure S4 Association between chronological age and estimated DNA methylation Age inferred by PhenoAge.** Black dots represent individuals. Red dashed line indicates model fit. Blue dashed line indicates identity line

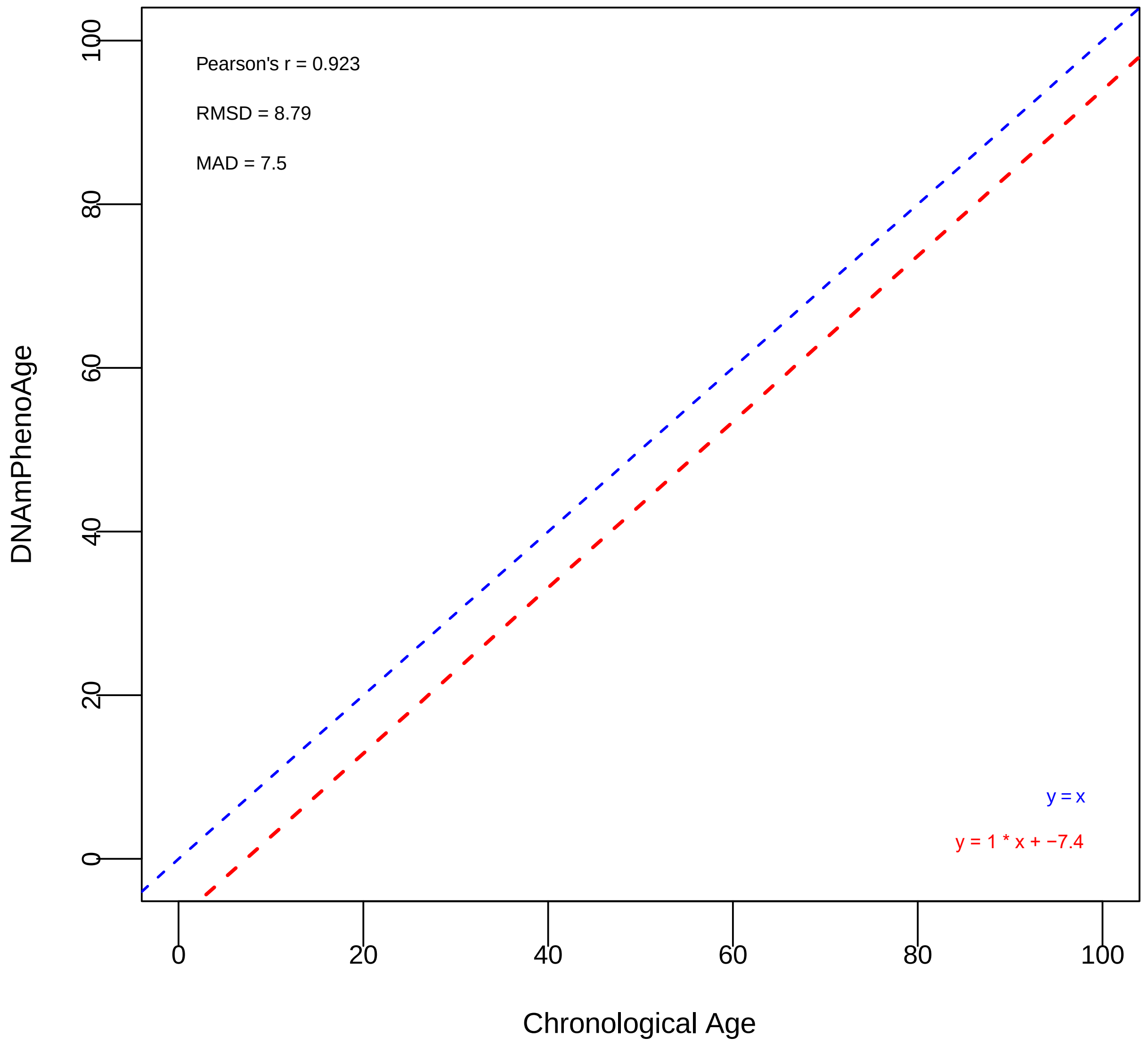

**Figure S5 Association between chronological age and estimated DNA methylation Age inferred by GrimAge.** Black dots represent individuals. Red dashed line indicates model fit. Blue dashed line indicates identity line

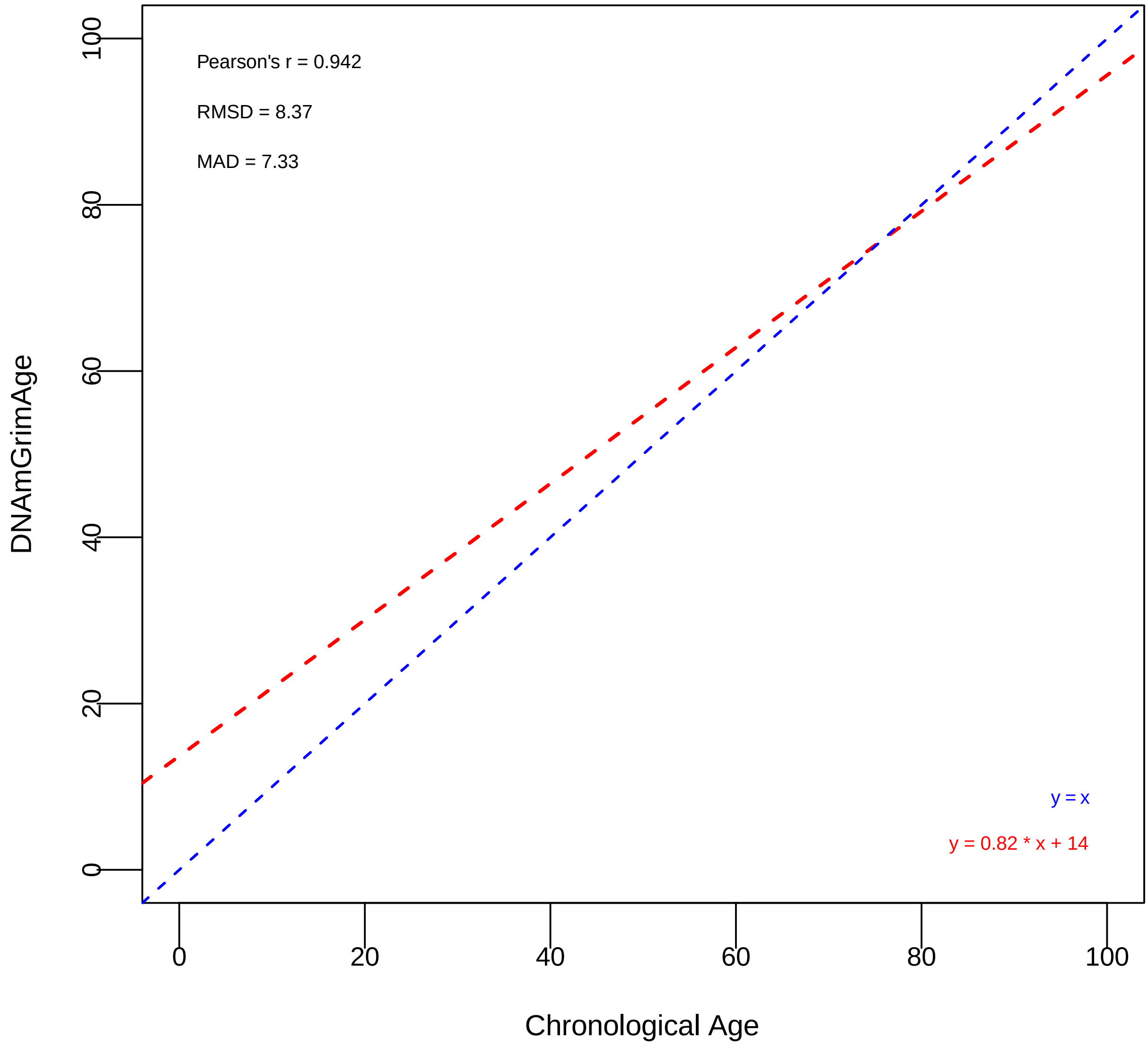

**Figure S6** Construction of the final sample

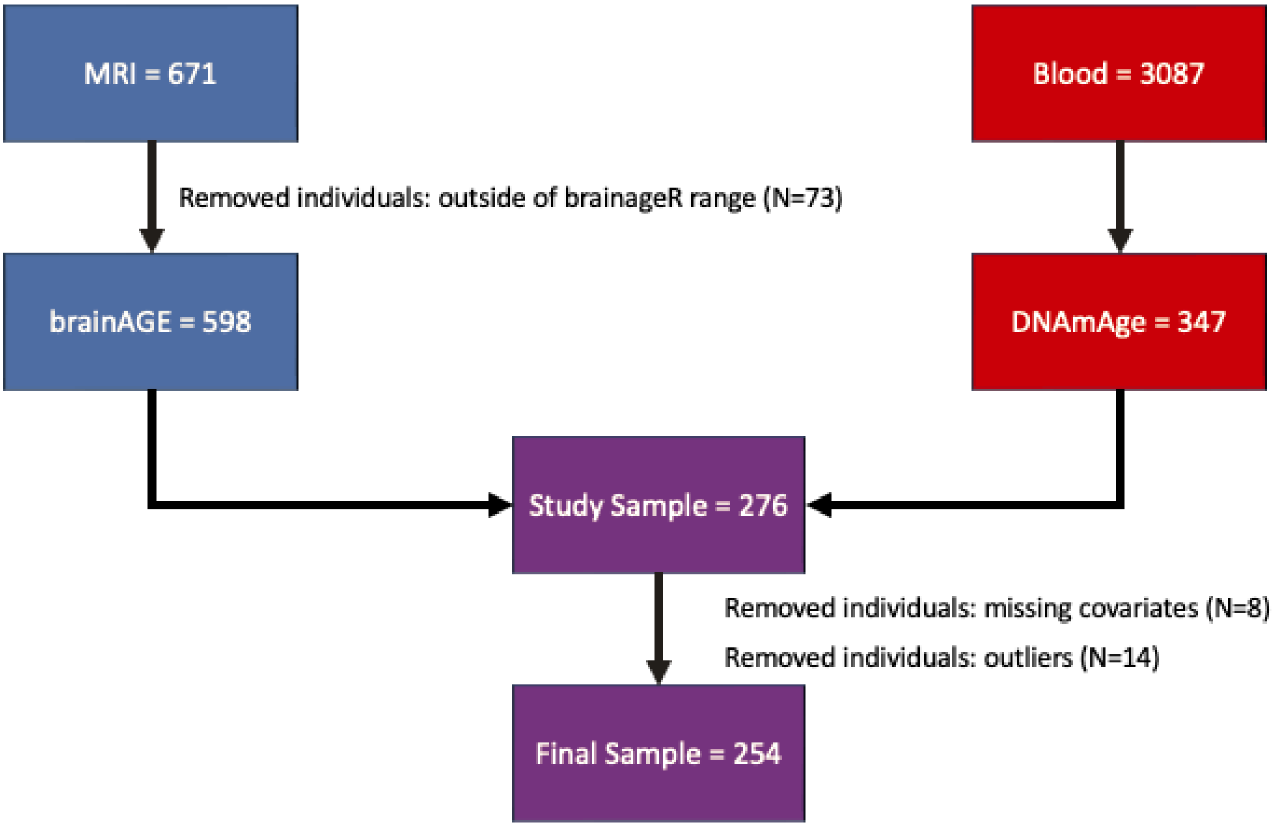

# References

1. Cole JH, Ritchie SJ, Bastin ME, et al. Brain age predicts mortality. *Mol Psychiatry*. 2018;23(5):1385-1392. doi:10.1038/mp.2017.62

2. McLachlan KJJ, Cole JH, Harris SE, Marioni RE, Deary IJ, Gale CR. Attitudes to ageing, biomarkers of ageing and mortality: The Lothian Birth Cohort 1936. *J Epidemiol Community Health*. 2020;74(4):377-383. doi:10.1136/jech-2019-213462

3. Teeuw J, Ori APS, Brouwer RM, et al. Accelerated aging in the brain, epigenetic aging in blood, and polygenic risk for schizophrenia. *Schizophr Res*. 2021;231:189-197. doi:10.1016/j.schres.2021.04.005

4. Peterson JA, Strath LJ, Nodarse CL, et al. Epigenetic Aging Mediates the Association between Pain Impact and Brain Aging in Middle to Older Age Individuals with Knee Pain. *Epigenetics*. 2022;17(13):2178-2187. doi:10.1080/15592294.2022.2111752

5. Zheng Y, Habes M, Gonzales M, et al. Mid-life epigenetic age, neuroimaging brain age, and cognitive function: coronary artery risk development in young adults (CARDIA) study. *Aging*. 2022;14(4):1691-1712. doi:10.18632/aging.203918

6. Faye Sanders, Vilte Baltramonaityte, Gary Donohoe, et al. Associations between methylation age and brain age in late adolescence. *bioRxiv*. Published online January 1, 2022:2022.09.08.506972. doi:10.1101/2022.09.08.506972

7. Phyo AZZ, Fransquet PD, Wrigglesworth J, Woods RL, Espinoza SE, Ryan J. Sex differences in biological aging and the association with clinical measures in older adults. *GeroScience*. 2024;46(2):1775-1788. doi:10.1007/s11357-023-00941-z

8. Eavani H, Habes M, Satterthwaite TD, et al. Heterogeneity of structural and functional imaging patterns of advanced brain aging revealed via machine learning methods. *Neurobiol Aging*. 2018;71:41-50. doi:10.1016/j.neurobiolaging.2018.06.013

9. Lu AT, Binder AM, Zhang J, et al. DNA methylation GrimAge version 2. *Aging*. 2022;14(23):9484-9549. doi:10.18632/aging.204434

10. den Braber A, van ’t Ent D, Cath DC, Wagner J, Boomsma DI, de Geus EJC. Brain activation during cognitive planning in twins discordant or concordant for obsessive-compulsive symptoms. *Brain J Neurol*. 2010;133(10):3123-3140. doi:10.1093/brain/awq229

11. Konijnenberg E, Carter SF, ten Kate M, et al. The EMIF-AD PreclinAD study: study design and baseline cohort overview. *Alzheimers Res Ther*. 2018;10(1):75. doi:10.1186/s13195-018-0406-7
